# Three years application of selective digestive decontamination in a mixed intensive care unit in a university hospital: impact on colonization, infection and antibiotic consumption

**DOI:** 10.1186/2197-425X-3-S1-A1009

**Published:** 2015-10-01

**Authors:** C Sánchez Ramirez, M Cabrera Santana, MA Hernández Viera, S Hípola Escalada, L Caipe Balcázar, N Sangil Monroy, A Bordes Benitez, F Artiles Campelo, P Saavedra Santana, S Ruiz Santana

**Affiliations:** Intensive Care Unit, Las Palmas de Gran Canaria, University Hospital of Gran Canaria Dr Negrín, Spain; Pharmacy Department, Las Palmas de Gran Canaria, University Hospital of Gran Canaria Dr Negrín, Spain; Microbiology Department, Las Palmas de Gran Canaria, University Hospital of Gran Canaria Dr Negrín, Spain; Mathematics and Informatcs Department, Las Palmas de Gran Canaria, University of Las Palmas de Gran Canaria, Spain

## Objectives

To prospectively evaluate the impact after 3 years of Selective Digestive Digestive (SDD) application on colonization, infection and antibiotic consumption in an ICU.

## Methods

This study was conducted in a 30-bed-medical-surgical ICU. All consecutive patients admitted to the from October 1, 2011 to September 30, 2014 expected to require tracheal intubation for longer than 48 hours were given SDD (SDD study group) with a 4-day course of intravenous cefotaxime, plus enteral colistin, tobramycin, nystatin in an oropharyngeal paste and in a digestive solution. Oropharyngeal and rectal swabs were obtained on admission and once weekly. Nosocomial infections were diagnosed by CDC criteria. We compared all patients admitted to ICU who acquired nosocomial ICU colonization and infection from October 1, 2010 to September 30, 2011 (non-SDD group) to SDD group. In both groups, categorical variables were summarized as frequencies and percentages and the continuous ones as means and standard deviations when the data followed the normal distribution or medians and interquartile ranges when they did not. The percentages were compared using the test of chi-square test or Fisher exact test, means with the t-test and medians with the Wilcoxon test for independent samples. Those variables that showed statistical significance in the univariate analysis were introduced in a multivariate logistic regression analysis. For each one of the acquired infections (catheter-related and other secondary bacteremias, pneumonia and urinary infections and antibiotic resistant bacteria (ARB) infection) the incidences per 1000 days of exposure in each cohort and the corresponding relative risks were obtained using the Poisson regression. Statistical significance was set at *p* ≤ 0.05. We also analized colistin and tobramycin resistant colonization and antibiotic consumption (Defined Antibiotics daily Doses (DDD)).

## Results

**Figure 1:**
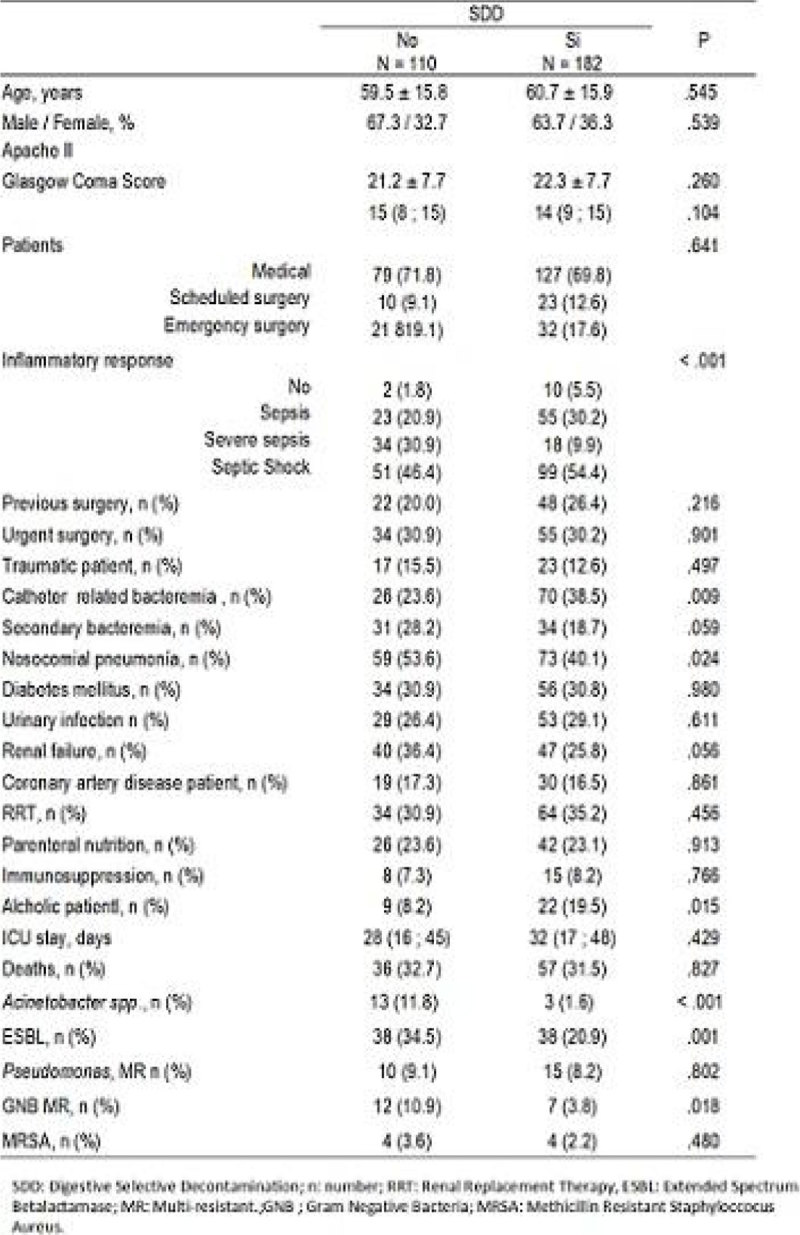
**Univariate analysis**.

**Figure 2:**
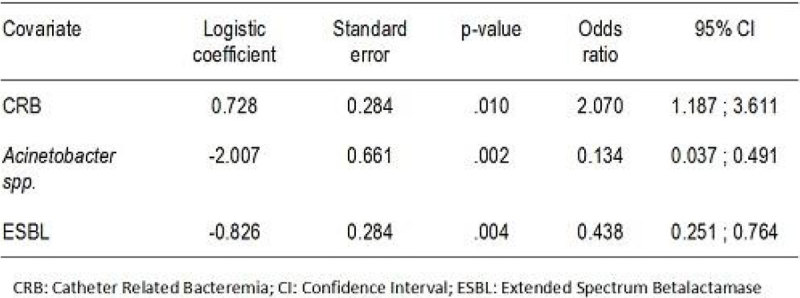
**Multivariate analysis**.

**Figure 3:**
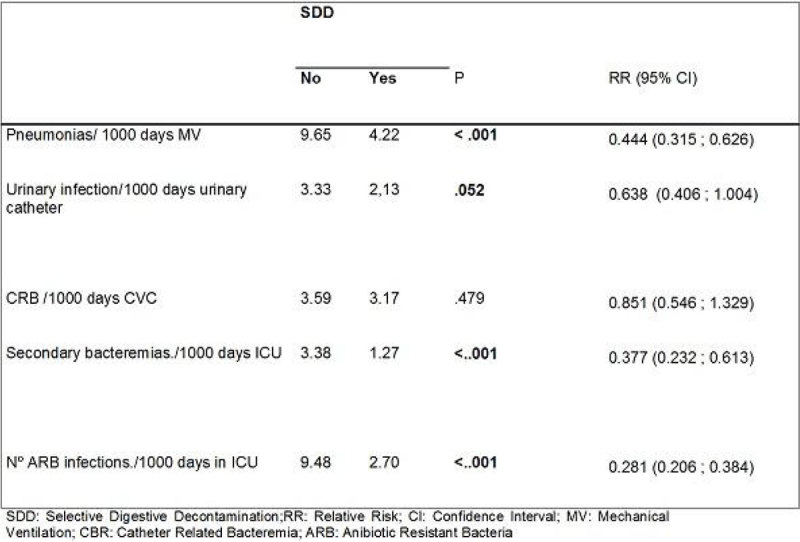
**Nosocomial infection rates**.

Results are shown in Figures 1, 2, 3.

There were no statistical significant differences between both groups in type of ICU admission or demographic data. Patients with SDD had significantly less ESBLs and *Acinetobacter spp*. We had also a significant reduction in nosocomial pneumonias, urinary tract infections and other secondary bacteremias and ARB rates in SDD group versus non SDD. There was no infections by Clostridium difficile. The exogenous infections were 84,2%. Colistin resistant colonization was 10,3% and tobramycin resistant colonization was 15,8% out of 253 samples of the studied patients. There was a decrease on the DDD/100 ICU stays during SDD.

## Conclusions

After 3 years applying SDD a significant reduction of infections by ESBL and *Acinetobacter* was observed. A significant decrease of nosocomial pneumonia, urinary infections and secondary bacteremias and ARB infections rates was shown. An antibiotic consumption reduction was shown compared to the non-SDD group. Colistin and tobramycin resistant colonization bacteria were also described.

